# The dental technician as a member of the hypodontia multidisciplinary team, with practical considerations for anterior restoration design

**DOI:** 10.1038/s41415-023-6331-6

**Published:** 2023-10-13

**Authors:** Stephen Ford, Martin P. Ashley

**Affiliations:** 41415431694001 Lead Dental Technician, University Dental Hospital of Manchester, Manchester, UK; 642685289736955458681 Consultant and MAHSC Honorary Professor in Restorative Dentistry and Oral Health, University Dental Hospital of Manchester, Manchester, United Kingdom

## Abstract

Most patients seeking treatment for hypodontia will require prosthetic replacement of their missing teeth. This will be in the form of dentures, bridges and implant restorations. As these are created by one or more dental technicians who supports the clinical team, a close working relationship between these colleagues is likely to improve the quality of treatment outcome. This interaction will usually occur towards the end of the patient's treatment process, when definitive restorations are prescribed. However, appropriately trained and experienced dental technicians should be involved throughout the patient's treatment process as an integral part of the multidisciplinary team approach to effectively manage these patients.

This paper describes the contribution of dental technicians to patient care with particular focus on communication between the restorative dentistry clinical team and the dental technician to improve the quality of anterior restorations. As missing maxillary lateral incisor teeth are a common presentation for this patient group, further technical detail relating to planning resin-bonded bridges for replacement of these teeth is included.

## Key points

- Dental technicians are integral members of the multi-disciplinary team, contributing to the high-quality treatment outcome.
- Hypodontia patients may require both interim and definitive appliances and prostheses at various stages of their treatment.
- Achieving an attractive and functional replacement for missing anterior teeth requires understanding of key design principles, by both the dental technicians and the clinical team.

## Introduction

Clinical management of hypodontia patients can be complex and lengthy, requiring contribution from various dental specialties. The dental technician should be an integral part of the multidisciplinary team and be involved throughout the patient's treatment process, providing hypodontia patients with both interim and definitive appliances and prostheses at various stages of their treatment.

Achieving an attractive and functional replacement for missing anterior teeth requires understanding of key design principles, by both the dental technicians and the clinical team.

## Orthodontic treatment

**Fig. 1:**
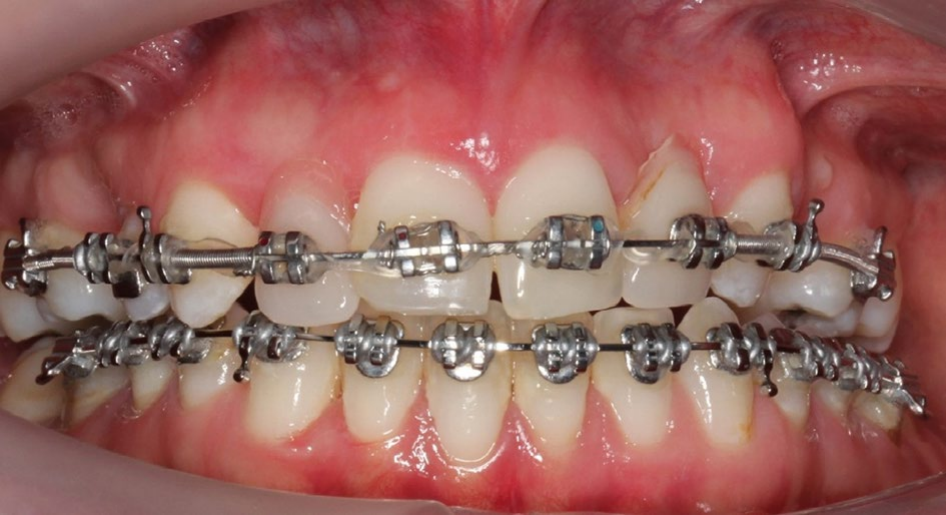
Orthodontic treatment for a hypodontia patient with missing maxillary lateral incisor teeth. Rather than have an unsightly gap during the treatment period, acrylic prosthetic teeth are added to the rectangular arch wire. Image courtesy of Dr Mariyah Nazir

A significant proportion of hypodontia patients will receive orthodontic treatment. The dental technician will be involved in fabrication of removable orthodontic appliances, usually used during the initial orthodontic treatment phase. In addition, if the creation of space significantly affects the patient's dental appearance, the dental technician can fabricate a small prosthetic tooth or teeth that can be connected directly onto the orthodontic appliance in the position of the missing teeth (Fig. 1).

**Fig. 2:**
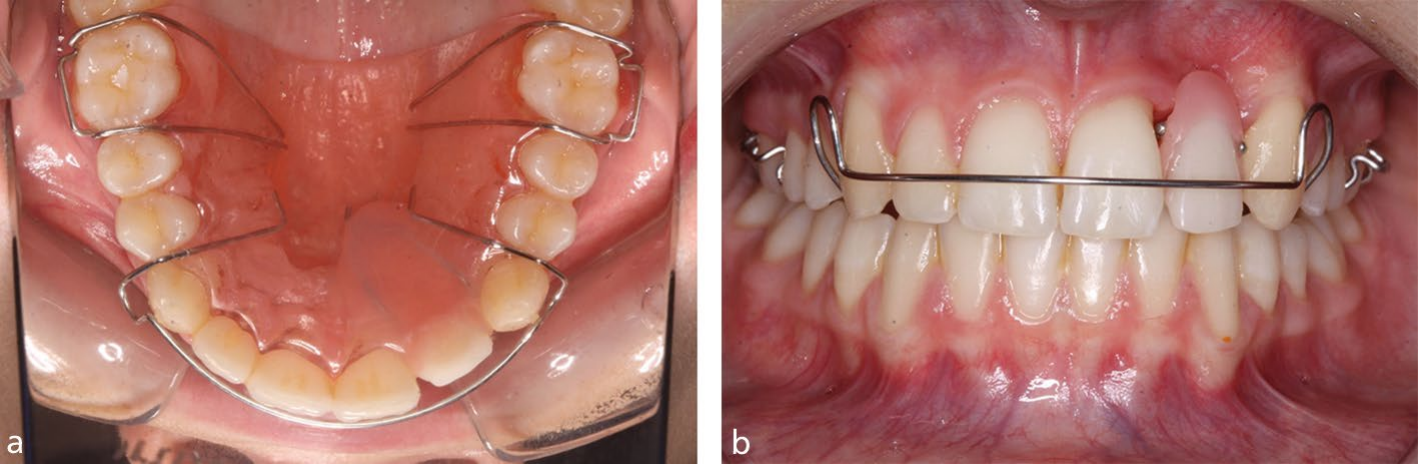
a, b) A modified Hawley retainer appliance to replace the missing maxillary lateral incisor tooth. Note the metal spurs to maintain the retracted position of the adjacent central incisor and canine teeth, in case the prosthetic tooth was lost

At the end of orthodontic treatment, the dental technician will be requested to provide a suitably designed orthodontic retainer appliance, often with a very short turnaround period, to ensure that the natural teeth remain in the desired position and also to suitably replace the missing teeth (Fig. 2).

## Removable restorations

**Fig. 3:**
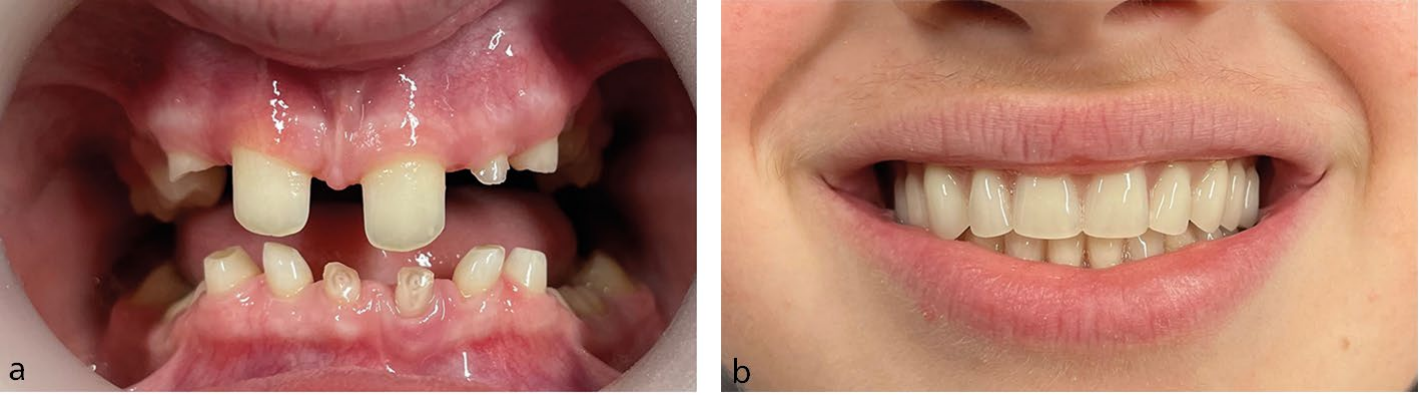
a, b) Removable complete overdentures as definitive restoration for a teenage hypodontia patient. These also serve in the design process for any future treatment the patient will require
- A radiographic stent, as part of the diagnostic stages of dental implant treatment, or as a surgical stent, to improve the accuracy of dental implant placement
- Provisional, interim restorations while other aspects of treatment, such as dental implant surgery, are completed
- Part of the design and treatment planning process required during the provision of fixed restorations.

A small number of hypodontia patients are missing so many teeth that even during childhood they can be provided with removable partial or complete dentures in order to improve their appearance, chewing ability and speech. However, removable prostheses are more commonly required after orthodontic treatment when the patient is older, as:

- Definitive restorations for replacement of missing teeth (Fig. 3)

## Interim fixed restorations

A small number of patients may benefit from having an interim fixed restoration during other treatment phases, such as either when additional, absolute anchorage is required during orthodontic treatment and a suitably restored dental implant is used, or an adhesive bridge is used during the period before dental implant surgery can be provided.

## Definitive fixed restorations

While some patients with hypodontia may not require orthodontic treatment, many do so in order to create spaces suitable for replacement of the missing teeth. The restorative dentist understands the clinical and technical dimensions required for restoring a space between natural teeth and by contributing to the orthodontic treatment plan, the precise development of the three-dimensional space can be achieved. The dental technician can subsequently make a more realistic replacement tooth of ideal proportions.

## The prescription for a fixed restoration

An equal partnership between clinician and dental technician is required to ensure a restoration is created, with suitable form and function, providing a reliable and attractive replacement for the missing tooth. In its simplest form, 'crown 46, shade B3' may possibly be acceptable instructions for a molar crown but 'RBB 12 and 13, shade A2' would certainly be inadequate for an anterior resin-bonded bridge (RBB). The more information that the clinician can contribute within the prescription, the more likely the dental technician will be able to create an acceptable restoration.

**Fig. 4:**
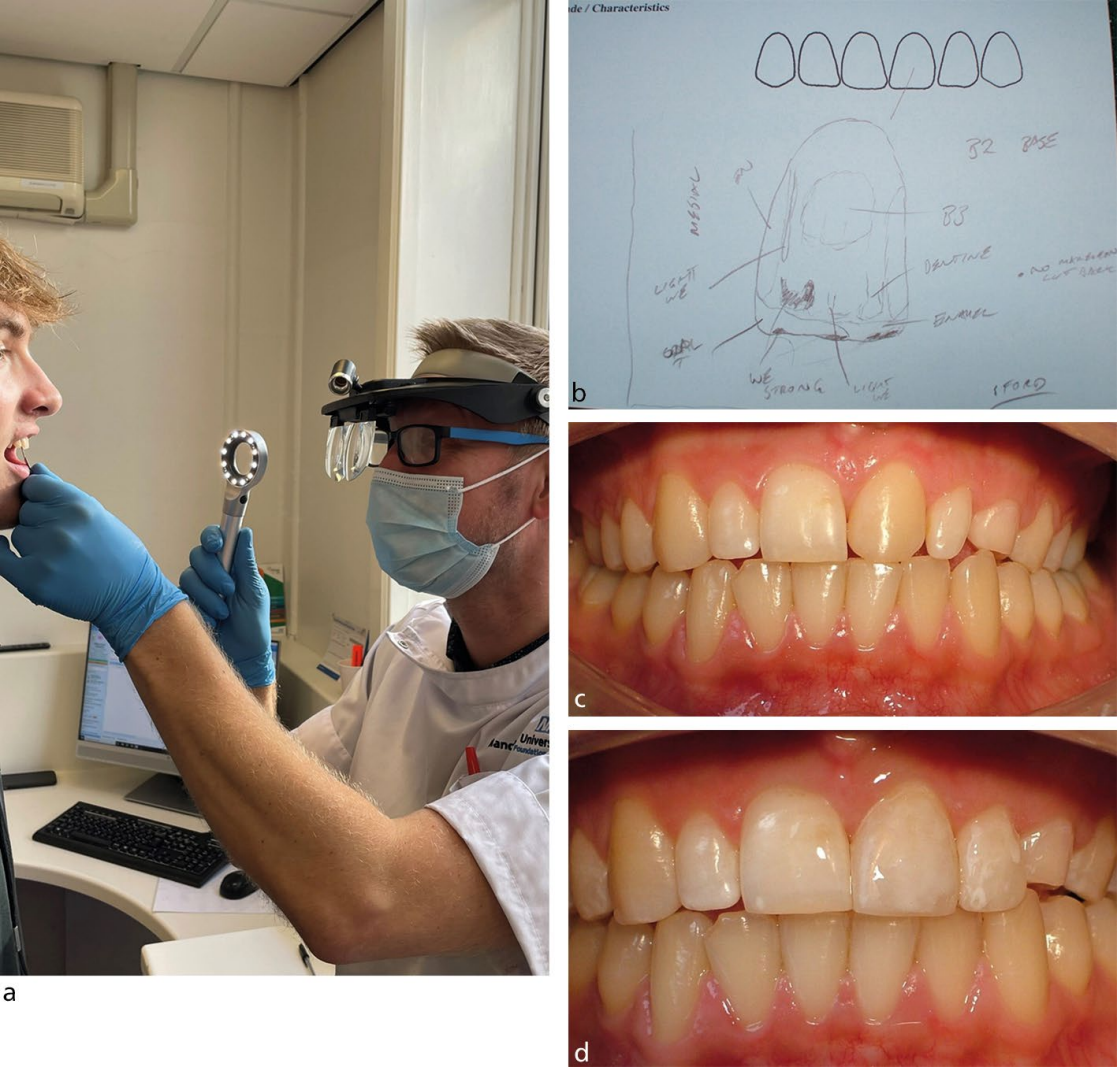
a, b, c, d) By meeting the patient, the dental technician has an opportunity to closely examine the dentition under ideal illumination conditions. A detailed tooth map records the subtle variations in shade and surface contours and enables the creation of acceptable restorations

The prescription is at least a written set of instructions that the dental technician requires. In addition, the clinician provides dental impressions or intra-oral scans, clinical photographs, occlusal records, and potentially, an articulator transfer bow. The photographs will display the extra-oral and intra-oral views, and by including the selected shade tabs in the close-up photographs, can also assist in demonstrating the natural dental appearance that is to be replicated. Further information can be shared by the clinician if they are able to discuss the case in person, or virtually with the dental technician during the fabrication stages. For more challenging clinical situations, it is useful for the dental technician to meet the patient and carry out a close examination of the dentition, particularly when selecting shades and surface characteristics for the restoration (Fig. 4).

## Diagnostic support

**Fig. 5:**
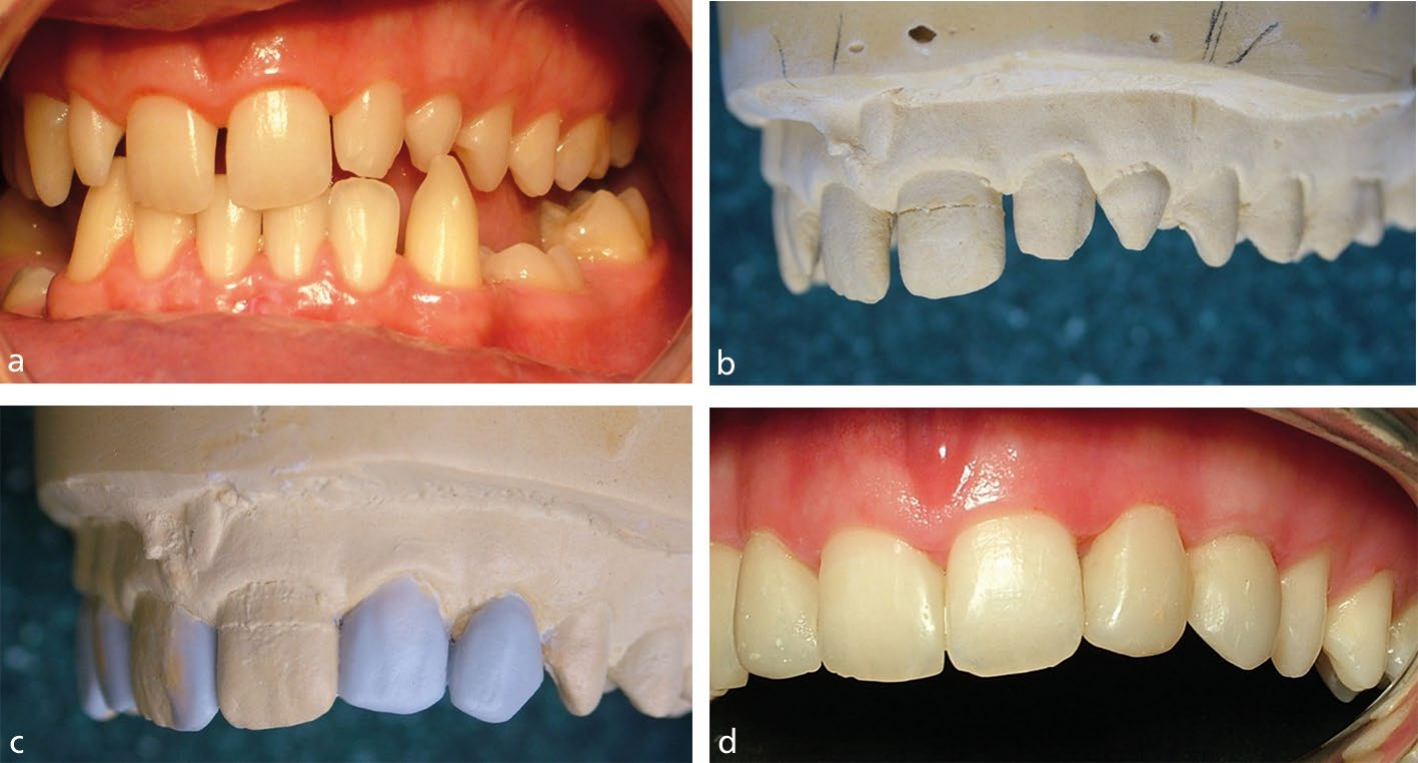
a) Patient affected by hypodontia seeking an aesthetic improvement to the small and spaced maxillary anterior teeth. b) Study cast of maxillary teeth. c) Study cast of maxillary teeth, with wax additions to clinician's prescription. d) Direct composite restoration of maxillary anterior teeth, guided by diagnostic casts

By modification of study casts to create diagnostic models, the dental technician can support and inform the clinician and patient in the planning of restorations (Fig. 5).

The 'wax-up' or 'mock-up' models can be both a visual guide to what is possible and can allow the creation of a clinical matrix.

## Designing a resin-bonded bridge

As well as simply identifying the tooth or teeth that will act as abutment(s) for the replacement teeth, the clinical team will consider several features of the space and the potential abutment teeth which will guide the restoration design.

Previous long-term studies^1^^,^^2^^,^^3^^,^^4^ have provided information about the ideal designs for an RBB. These have guided the further development of clinical techniques and prosthetic designs, with the intention of improved outcomes.^5^

## Abutment teeth

Any tooth adjacent to the space should be considered as a potential abutment tooth.

**Fig. 6:**
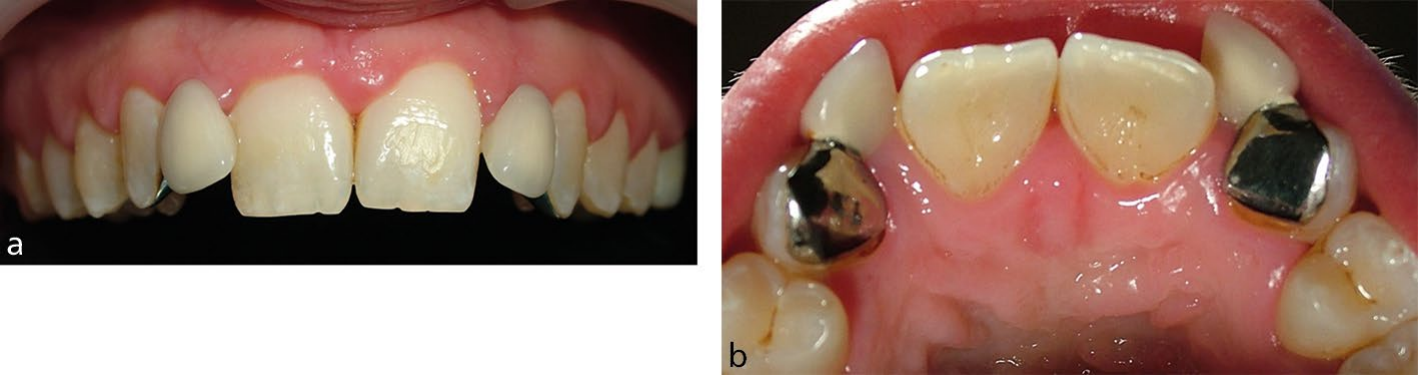
a, b) Missing maxillary lateral incisor teeth replaced by RBBs from the adjacent maxillary canine teeth. Without adequate orthodontic retention, relapse of the canine teeth by outward rotation, potentially with retroclination of the central incisor teeth, leads to exaggerated movement of the bridge pontics into unaesthetic positions

Given the potential for increased translucency of the edges of maxillary incisor teeth compared to canine teeth, clinicians may consider canine teeth to be more suitable as abutment teeth for lateral incisor RBBs. Canine teeth that have been orthodontically repositioned are likely to rotate when relapsing. Therefore, a cantilever lateral incisor bridge attached to a canine will potentially move significantly as the canine relapses (Fig. 6).

**Fig. 7:**
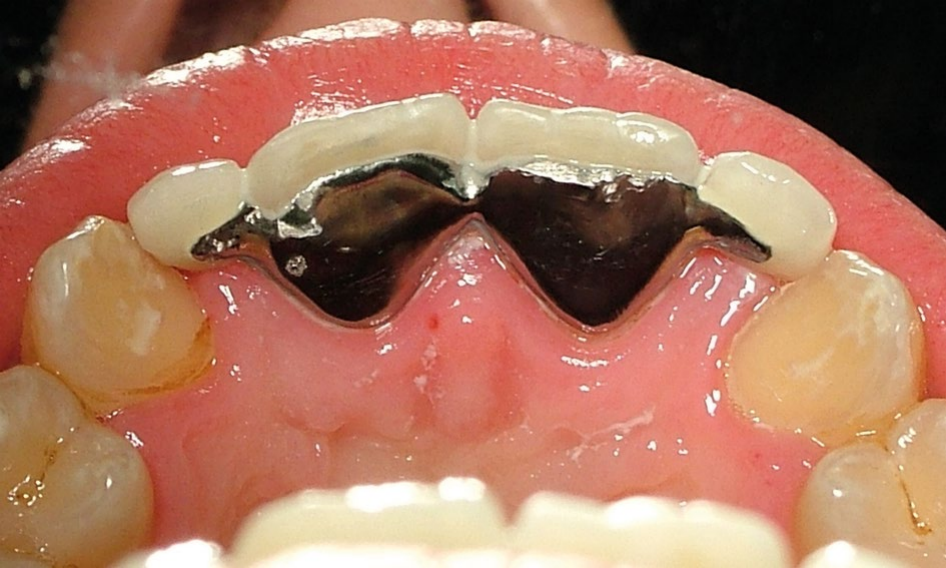
The same patient as illustrated in Figure 6, retreated with an RBB secured to both maxillary central incisor teeth. Rather than pursue further orthodontic treatment again, the desired improvement in dental appearance achieved by provision of a new bridge was completed in three appointments

Alternatively, the option of providing a suitably designed bridge from both maxillary central incisor teeth to replace both lateral incisor teeth as a single restoration can be favourable and has a relatively high success rate^4^(Fig. 7). This design also ensures that the central incisor teeth do not relapse significantly, particularly if the orthodontic treatment had closed a diastema between these two teeth.

## Bridge wing design

**Fig. 8:**
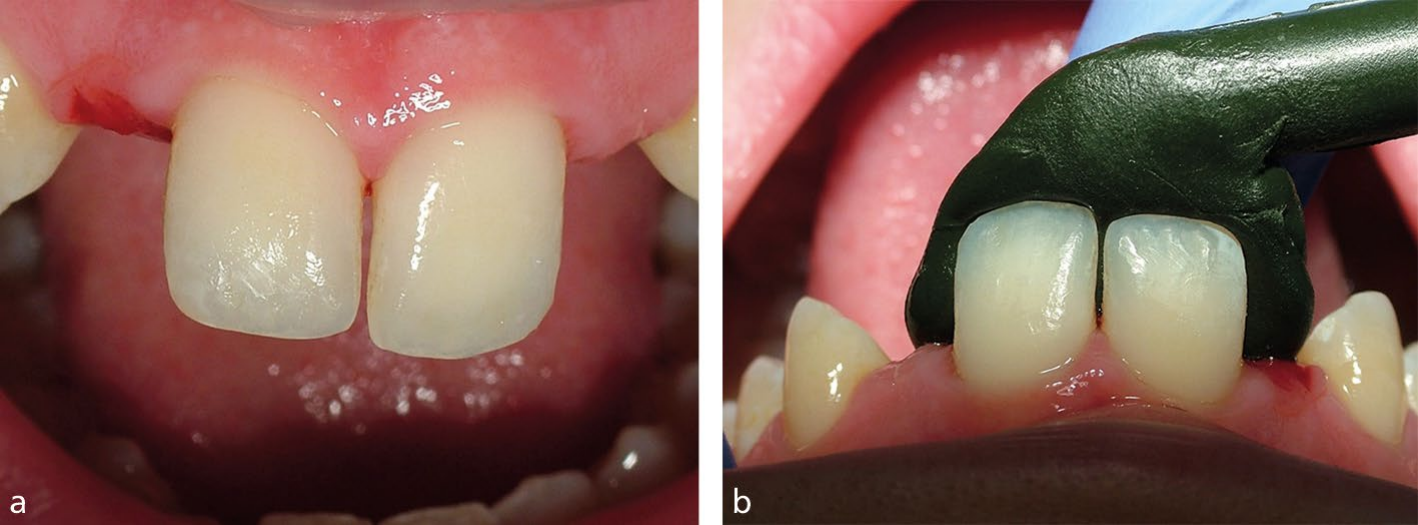
a, b) The translucency of the incisal edges can be assessed by moulding a dark material, such as greenstick impression compound, to the palatal surfaces of the abutment incisor teeth. Once moulded, it is advisable to wet the greenstick to enhance the interface between moulding and tooth surface, which increases the apparent translucency to similar that would occur if the metal bridge wings inappropriately covered the same area

The retaining wings should cover the maximum amount of enamel surface area, but this will usually cause a problem with grey shine-through in the translucent incisal edge. To avoid this, the dental technician is instructed to only extend the retaining wing to an identified position, such as 2 mm from the incisal edge. The clinician can precisely assess this incisor translucency by moulding a dark material against the palatal surface, such as green-stick impression compound (Henry Schein Ltd, UK) (Fig. 8). It is unusual for the incisal translucency to extend more than 2 mm from the incisal edge.

**Fig. 9:**
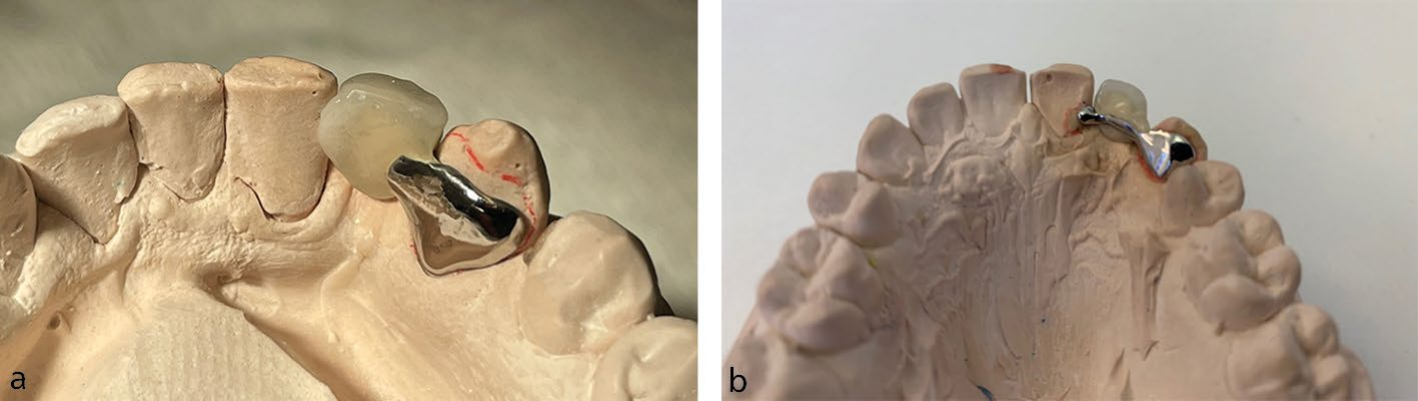
a, b) The mesial surface of the lateral incisor tooth pontic is extended to just overlap the distal surface of the adjacent incisor tooth, reducing the potential for rotational relapse of the canine abutment tooth. The extension is not bonded to the central incisor tooth

If the condition of the central incisor teeth is unsuitable for use as abutment teeth, or if the bonding of a fixed orthodontic retainer wire in this area has compromised the enamel surface for successful bridge bonding, it may actually be necessary to use a canine tooth to support the bridge. In order to reduce the potential for rotational relapse which would affect the lateral incisor pontic, the mesial surface contact area of the pontic can be modified to just overlap onto, but not bonded to, the palatal surface of the adjacent central incisor tooth. This should be sufficient to prevent outward rotation but still allows adequate cleaning of the central incisor to reduce the risk of caries (Fig. 9).

**Fig. 10:**
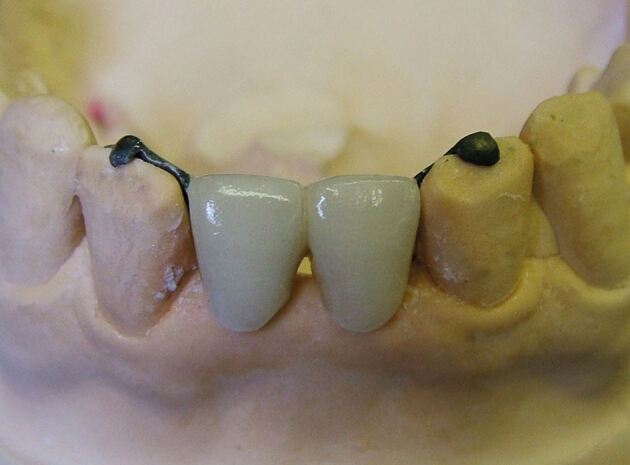
RBB with incisal hooks on abutment teeth

During fabrication of the bridge, the dental technician may design a small incisal hook as an extension of the metal wing that fits onto the incisal edge of the supporting tooth on the working model, to aid retention during the porcelain layering stages. This hook can be left *in situ* to provide some additional stability during the clinical cementation process (Fig. 10). It is then removed with a small diamond bur when the cementation is completed.^5^ Alternatively, as there is a risk that the hook prevents accurate seating of the wing onto the palatal enamel, the incisal hook can be removed after trial fit but before cementation, if the clinician is confident with the visual assessment of the fit accuracy of the bridge.

## The position of the pontic cervical margin

In order to create symmetry for the cervical margins of the lateral incisor pontic, it is usually appropriate to create a restoration for which the cervical margin matches that of the contralateral natural tooth. If both lateral incisor teeth are to be replaced by the restoration, the cervical margins are generally 1 mm below a line visualised from the zenith of the adjacent central incisor and canine teeth.

As the patient will be required to maintain an adequate standard of dental hygiene around the bridge each day, the pontic design must allow either a narrow interdental brush or dental floss to pass into the embrasure area.

As the pontic site is generally underdeveloped and concave compared to the ridge form above the adjacent central incisor and canine teeth, a decision is required for how the restoration is designed in relation to contact with the ridge. If this is done incorrectly, there can be an unattractive shadow above the bridge pontic. In order to do this, the dental technician can be instructed to alter the pontic site on the model, so that when the bridge is fitted, there is light displacement of the tissues, allowing an appearance of the pontic emerging from within the tissues in the same ways a natural tooth would. For more challenging aesthetic cases, pontic site development surgical techniques have been developed.

## The incisal edge and the occlusal surface

The correct position of the incisal edge can usually be determined by aiming for symmetry with the contralateral tooth. If this is not possible, the incisal edge is usually 1 mm higher than a virtual line drawn between the adjacent incisal edge and canine tip. Care should be taken to consider the position of the opposing lower teeth to reduce the potential for unwanted incisal contacts. A good orthodontic outcome can ensure that the position of the opposing lower teeth is favourable and that an incomplete overbite is created, with sufficient space for the bridge pontic and wing(s). On occasions, the opposing lower teeth are not ideally positioned, and the restorative dentist will need to confirm whether the opposing lower teeth can be adjusted at the bridge fit appointment to ensure the contacts are favourable. Alternatively, the bridge can be cemented 'high' with intentional contact against the opposing teeth. It is hoped that all the affected teeth adapt and re-establish normal occlusal contacts before the bridge is overloaded and debonds.

## Unfavourable space for a pontic

On occasions, the three-dimensional space for a replacement tooth is not ideal. This can be either despite the best outcome achieved after orthodontic treatment, or because orthodontic treatment is either refused by the patient or not offered by the clinical team. In these situations, restoration of the space by replacing the missing teeth requires the dental technician and restorative dentist to work closely together to achieve an acceptable, if perhaps compromised, outcome.

Most of the compromised situations involve a space that is either too wide or too narrow to allow a restoration of ideal proportions. Therefore, variations in the pontic size, modification of adjacent teeth, and acceptance of residual spaces can all be considered to achieve an acceptable, if not ideal, outcome. Such a treatment approach should be planned carefully and an intra-oral trial may be of use to ensure the patient accepts the proposed solution.

The vertical space available for replacing the missing tooth should be assessed in both static and dynamic positions to ensure the restorations, especially RBB pontics, will not be overloaded, leading to the potential for early and repeated restoration failure. This situation is usually avoided by a good orthodontic outcome. Occasionally, it is necessary to alter the incisal edges of the opposing natural teeth to eliminate these dynamic contacts. The patient should be informed of the proposed adjustments before the restoration has been placed, and ideally during the restorative dentistry treatment planning process, to maintain their understanding and agreement for the treatment process.

## Conclusion

As many patients with hypodontia require some form of orthodontic appliance or dental restoration during their treatment, the dental technician is an essential part of the multi-disciplinary team. Close communication and engagement of clinical and technical colleagues throughout the often lengthy treatment period contribute to a better-quality outcome. Achieving an attractive and functional replacement for missing teeth requires the clinician to provide much more information to their dental technician colleague than simply stating a shade and which restoration is required. The process starts earlier in the treatment process and the dental technician, as a key member of the hypodontia MDT, will support their clinical colleagues at various treatment stages.

Replacing missing anterior teeth and restoring other anterior teeth is usually a challenge, requiring clinicians and dental technicians to collaborate together in partnership. By understanding each other's work, ideal three-dimensional tooth positioning and space creation between the natural teeth can enable the use of restorations to achieve acceptable aesthetic and functional outcomes.
